# Creating a list of word alignments from parallel Russian simplification data

**DOI:** 10.3389/frai.2022.984759

**Published:** 2022-09-12

**Authors:** Anna Dmitrieva, Antonina Laposhina, Maria Yuryevna Lebedeva

**Affiliations:** ^1^Faculty of Arts, University of Helsinki, Helsinki, Finland; ^2^Language and Cognition Laboratory, Pushkin State Russian Language Institute, Moscow, Russia

**Keywords:** lexical simplification, lexical substitution, vocabulary list, monolingual word alignment, Simple Russian, Russian as a foreign language

## Abstract

This work describes the development of a list of monolingual word alignments taken from parallel Russian simplification data. This word lists can be used in such lexical simplification tasks as rule-based simplification applications and lexically constrained decoding for neural machine translation models. Moreover, they constitute a valuable source of information for developing educational materials for teaching Russian as a second/foreign language. In this work, a word list was compiled automatically and post-edited by human experts. The resulting list contains 1409 word pairs in which each “complex” word has an equivalent “simpler” (shorter, more frequent, modern, international) synonym. We studied the contents of the word list by comparing the frequencies of the words in the pairs and their levels in the special CEFR-graded vocabulary lists for learners of Russian as a foreign language. The evaluation demonstrated that lexical simplification by means of single-word synonym replacement does not occur often in the adapted texts. The resulting list also illustrates the peculiarities of the lexical simplification task for L2 learners, such as the choice of a less frequent but international word.

## Introduction

Lexical simplification is one of the main strategies to make text easier to understand for L2 learners. The key subtask here is to find suitable candidates for replacing a complex word with a simpler synonym. This study explored the potential of monolingual word aligners for the development of a list of possible lexical substitutions. This is a special word list where each “complex” word has an equivalent “simpler” (shorter, more frequent, modern, international) synonym. This list was compiled automatically and post-edited by experts. After that, we analyzed the contents of the list and compared the words in it to the existing standardized lexical minima for different levels of Russian language proficiency and a frequency dictionary. Such parallel lists of words and their simpler alternatives can be used for text simplification purposes, for example, in rule-based simplification tools and in lexically constrained decoding for neural machine translation models. Moreover, it is a valuable source of information for curriculum and educational material creators.

Currently, there is not much data that can be used for Russian lexical simplification. The main source of information about lexical complexity and word usage is CEFR-graded vocabulary lists for learners of Russian as a second language (L2; Andryshina and Kozlova, 2012, 2015; Andryshina, 2017a,b). There are separate lists for each level of language proficiency from elementary to advanced. However, these lists do not provide information about synonym/hypernym/hyponym relationships between words. Conversely, while there are dictionaries of synonyms, they do not guarantee that a given synonym is simpler than a synonymized word. Therefore, in order to create, for instance, a reliable lexical simplification tool, a specialized word list would be needed.

## Related work

Most lexical simplification models involve replacing complex words or phrases with simpler ones. The source of data on the complexity of a word here can be formal text characteristics, e.g., word frequency or length (Shardlow, 2013), and the complexity ranking of words by native or non-native speakers (Maddela and Xu, 2018). The other source of data can be parallel corpora of original and simplified versions of a text, which illustrate the natural process of text adaptation. Parallel monolingual simplified corpora are essential both for extracting the simplification rules (Horn et al., 2014) and for evaluating the quality of unsupervised models (Qiang et al., 2019). The main difficulty of this method lies in the need to match original text fragments with simplified versions. The aligning process involves matching the corresponding parts of the original and simplified parts of a text at the paragraph, sentence, or individual word levels. Thus, monolingual word alignment aims to align words or phrases with similar meanings in two sentences that are written in the same language (Lan et al., 2021). Historically, word alignments have been used in tasks such as statistical machine translation and annotation transfer (Östling and Tiedemann, 2016), and today monolingual alignments can be useful for improving the interpretability in natural language understanding tasks, improving model performance for text-to-text generation tasks, and analyzing human editing operations (Lan et al., 2021). One of the text-to-text generation tasks that utilize lists of monolingual word alignments is lexical simplification.

There are not many tools suitable for monolingual language alignment of regular and simplified texts. However, many word alignment instruments have been created for parallel texts in different languages. Statistical systems such as GIZA++ (Och and Ney, 2003) or fast_align (Dyer et al., 2013) have been widely used for a long time; however, neural tools have also recently gained popularity. Neural network-based instruments can take advantage of large-scale contextualized word embeddings derived from language models multilingually trained on monolingual corpora (Dou and Neubig, 2021). Recently, neural tools specifically for monolingual alignment have started to appear (Lan et al., 2021), but so far no instruments have been developed for Russian.

Lexical simplification has proved to be one of the main text simplification strategies for the Russian second language learning purposes (Sibirtseva and Karpov, 2014, p. 25; Dmitrieva et al., 2021). It has also been shown that lexical substitution is an effective text adaptation strategy for children with reading disabilities (Zubov and Petrova, 2020). However, for the Russian language, attempts to automated lexical substitution are rare. In one study (Dmitrieva, 2016), lexical simplification is performed on Russian data by means of synonym replacement. The author created a list of synonym pairs for this purpose, where the target words are taken from the CEFR vocabulary lists and the source words were obtained from a dictionary of synonyms. The list is said to contain around 8,000 synonym pairs. However, the word pairs in this list were not taken from real parallel texts, which precludes the possibility of studying them as actual editing operations performed during text adaptation. This study aims to fill this gap and check the potential of the automated development of a list of candidates for lexical substitution based on a parallel corpus of original and adapted texts in Russian.

## Data

We use a parallel Russian simplification dataset to create a word list called RuAdapt^1^ (Dmitrieva et al., 2021). It has both paragraph-aligned and sentence-aligned versions, but we chose the sentence alignments for better performance of the automatic word alignment software. RuAdapt has three subcorpora with texts of different genres, all of which have been simplified by experts in teaching Russian as a foreign language. For this stage, we chose the adapted fiction books subcorpus, for it is the largest in the dataset. It includes 24,232 pairs of sentences taken from 93 texts. In total, there are 376,432 tokens in the original sentences and 285,190 tokens in their adapted equivalents. Examples of source (1) and target (2) data are shown below.

(1) К утру *A*нна задремала, сидя в кресле, и когда проснулась, то уже было бело, светло, и поезд подходил к Петербургу.
  /By morning, Anna dozed off, sitting in an armchair, and when she woke up, it was already white, light, and the train was approaching Petersburg./
(2) К утру *A*нна наконец заснула, а когда проснулась, уже было светло и поезд подходил к Петербургу.
  */*By morning, Anna finally fell asleep, and when she woke up, it was already light and the train was approaching Petersburg./

Each sentence pair in RuAdapt has a cosine similarity score that was assigned during automatic alignment by the CATS alignment tool (Štajner et al., 2017). For the purposes of this project, we chose 15,156 sentence pairs with a cosine similarity lower than 0.99 but higher than 0.31. These thresholds were chosen empirically: We wanted to omit not only pairs that are too different, since they most likely will not have many correct single-word alignments, but also nearly identical pairs.

## Alignment

For this project, we used one statistical aligner and one neural aligner. Aligning pairs of regular and simplified sentences can be easier and harder than aligning translations at the same time: On the one hand, the sentences are monolingual, but on the other hand, the sentence length often does not match and many words might be omitted. Therefore, we decided to use different aligners and compare the results. Before alignment, we did not lemmatize the sentences, because, to the best of our knowledge, the impact of lemmatization on monolingual alignment of different sentences has not yet been studied in detail. Also, as will be discussed below, some linguistic phenomena that we are interested in can be lost during lemmatization.

Eflomal (Efficient Low-Memory Aligner) is a system for efficient and accurate word alignment using a Bayesian model with Markov Chain Monte Carlo (MCMC) inference. It is based on the efmaral tool (Östling and Tiedemann, 2016), but has advantages such as lower memory costs. According to the performance comparison on the project's GitHub page, eflomal shows a lower alignment error rate than efmaral and fast_align on language pairs such as English–French and English–Hindi.

Similarly to other statistical aligners, eflomal requires a substantial amount of parallel data to train on. We decided to use pairs of paraphrases in Russian, since this type of monolingual parallel data is much easier to obtain than simplification data. We obtained an additional dataset of around 2.5 mil. paraphrases from Opusparcus (Creutz, 2018) and ParaPhraserPlus (Gudkov et al., 2020) and used it for training purposes.

Eflomal^2^ outputs Pharaoh-format alignments, where a pair of numbers *i*-*j* indicates that the *i*-th word source sentence corresponds to the *j*-th word of the target sentence. In order to obtain word-to-word alignments, a dedicated instrument from the Natural Language Toolkit (NLTK) called phrase_based^3^ was used. This is a phrase extraction algorithm that extracts all consistent phrase pairs from a word-aligned sentence pair, meaning that it is also possible to obtain phrase-to-word and phrase-to-phrase alignments. However, in this study we limited ourselves to single word pairs.

Another word alignment tool that we used is awesome-align (Aligning Word Embedding Spaces Of Multilingual Encoders), a tool that can extract word alignments from multilingual BERT and allows users to fine-tune mBERT on parallel corpora for better alignment quality (Dou and Neubig, 2021). Although it can be finetuned, no large training corpus is required prior to alignment, so we used the aligner as is. Awesome-align shows lower alignment error rates than eflomal on language pairs such as German–English and French–English.

**Figure 1:**
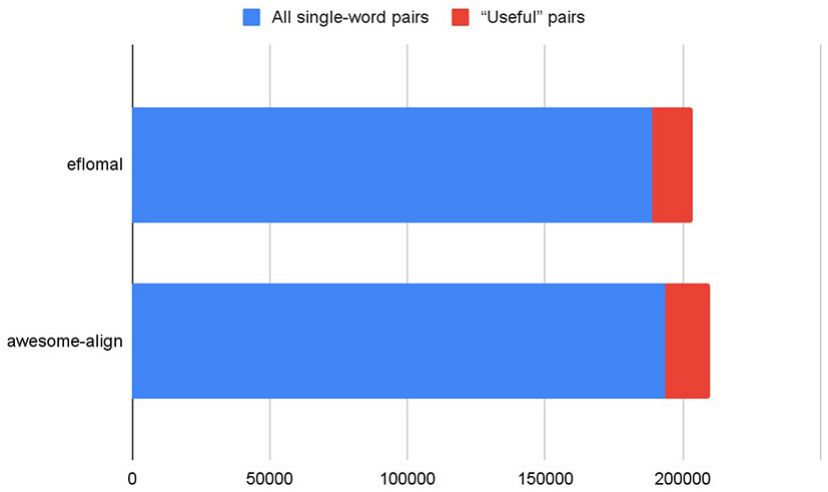
The percentage of “useful” pairs among all pairs.

The initial alignment results are shown in Table 1. As can be seen, many single-word pairs were obtained, but most of them were identical words. The percentage of “useful” pairs is in fact rather low, as is also shown on Figure 1. A “useful” pair of words is a pair that can potentially be included in the list of word alignments. In such pairs of words, the source word and target word are different and there is no noise (punctuation instead of words, etc.).

**Table 1:** Alignment statistics.

**Statistic**	**Eflomal**	**Awesome-align**
All single word pairs	188,706	193,778
Pairs consisting of different words, cleaned from noise	19,687	22,767
Unique pairs	14,807	15,989
Unique pairs in common	8,403	

Considering that the two aligners produced 8,403 identical pairs, there were 22,393 pairs in all at the end of the alignment process. However, these pairs still contained noise and non-synonymic pairs, which made it clear that human editing would be needed.

## Expert editing

In order to edit the word lists, 18 human editors were asked to check the 22,393 word pairs obtained on the previous step. All of the editors are students and/or specialists in teaching Russian as a foreign language from [institution name removed for anonymity]. Each pair was checked by at least two different editors.

Editors were asked to give each word pair a score of 0, 1, or 2 according to the following instructions:

- Score 0 is given to:

- noisy pairs (non-synonyms);
- pairs consisting of the same word or different forms of the same word.

- Score 1 is given to:

- pairs that can only be considered synonyms in a certain context;
- different words with the same root (e.g., звать/позвать);
- synonyms that are presented by different parts of speech.

- Score 2 is given to:

- pairs that are considered synonyms in most contexts;
- pairs where the source word is an older form of the target word (e.g., кофий/кофе).

A score of 2 is supposed to indicate that a pair can be included in the word alignment list; score 0 indicates the opposite. Score 1 is given in cases of uncertainty that may be included in the list or preserved for future studies. It is important to note that we are aiming to evaluate the “usefulness” of the pair for the word list, not the alignment quality.

During the first stage of editing, the editors worked with 14,807 word pairs produced by eflomal. In the second stage, there were only 7,586 pairs produced by awesome-align left to post-edit, since the two alignment instruments produced 8,403 identical pairs. In an attempt to further ease the editors' work, we tried to eliminate the words that had at least one same root, since they would not receive a score 2 and most in fact would receive 0 (being different forms of the same word). We used the NeuralMorphemeSegmentation tool^4^ (Sorokin and Kravtsova, 2018) to split the words into roots and affixes. However, there were not many word pairs excluded that way: This strategy detected 796 pairs, and after manual check 775 pairs were excluded, leaving the editors with 6,811 pairs to post-edit.

At the end of the post-editing process, only 2,336 pairs received a score of 2 from at least one editor, and 5,110 pairs received a score of 1 from at least one editor. We used Cohen's kappa score to measure inter-annotator agreement, yielding a score across all documents of 0.42, which is interpreted as indicating a moderate degree of agreement (McHugh, 2012). It is evident that in many cases deciding on a score was difficult even for humans.

Since we are mostly interested in pairs with a score of 2, we arranged a second evaluation for the pairs that received a score of 2 from at least one editor. A third expert evaluated 2,336 pairs and gave each a resulting score, resulting in 1,409 unique pairs with a score of 2, 1,755 pairs with a score of 1 (pairs that received 1 from both editors and pairs that received 1 during expert evaluation), and 14,493 pairs with a score of 0 (pairs that received 0 from both editors).

Out of the 1,409 pairs with a score of 2, 1,349 were obtained from awesome-align alone or both awesome-align and eflomal, and 1,197 were obtained from eflomal or both awesome-align and eflomal. That means that 9.11% of awesome-align alignments and 8.08% of eflomal alignments received a score of 2, which are both rather small percentages. Since the scores do not reflect the alignment quality directly, they does not illustrate the aligners' efficiency, but rather give an idea of how many single-word alignments will end up being synonyms.

The resulting list^5^ contains 1,409 pairs of word forms and their simpler synonyms approved by experts with 1,134 unique source lemmas and 811 unique target lemmas. The choice of classical literature as a data source leaves its mark on the types of lexical substitution: e.g., the list contains examples of replacing an archaic grammatical form of a word with a modern one (e.g., простою [simple ADJ+GEN, archaic] → простой [simple ADJ+GEN]), or replacing an obsolete word with a modern synonym (e.g., особливый [special, archaic] → отдельный [special]). However, most of the list presents more universal types of lexical simplification, such as replacing a word with a more neutral and frequent analog (e.g., умолять [to beg] → просить [to please]), use of hypernyms (e.g., соловьи [nightingales] → птицы [birds]), or the removal of subjective evaluation suffixes (e.g., деревенька [village+diminutive suffix] → деревня [village]).

## Word list evaluation

After obtaining the word lists, we examined the vocabulary that they contain. We were interested in pairs that received a score of 2 in the final evaluation. We first compared the word pairs to CEFR-graded vocabulary lists. Our hypothesis was that in a given pair, the source word is supposed to have a higher grade level than the target word (for example, an A2 level word should be replaced with an A1 synonym).

The obtained list evaluation included both the comparison of word pairs in special graded vocabulary lists and its general frequency. The most evident way to check if the target word is simpler in terms of Russian as a foreign language proficiency is to compare the first occurrences of source and target words in special vocabulary lists graded by the Common European Framework of Reference for languages (CEFR) levels. Our hypothesis is that the source word is supposed to have a higher grade level than the target word (e.g., a C1 word бормотать [to mutter] should be replaced with an A1 synonym говорить [to tell]).

Before comparing the word pairs to the frequency dictionary and CEFR vocabulary lists, we lemmatized them using the Stanza Python library^6^ (Qi et al., 2020). We used the default model, which was trained on Syntagrus (Droganova et al., 2018). We did not lemmatize just the words from the pairs, but the corresponding sentences and extracted the necessary lemmas from them, since the context can be important for correct lemmatization.

**Figure 2:**
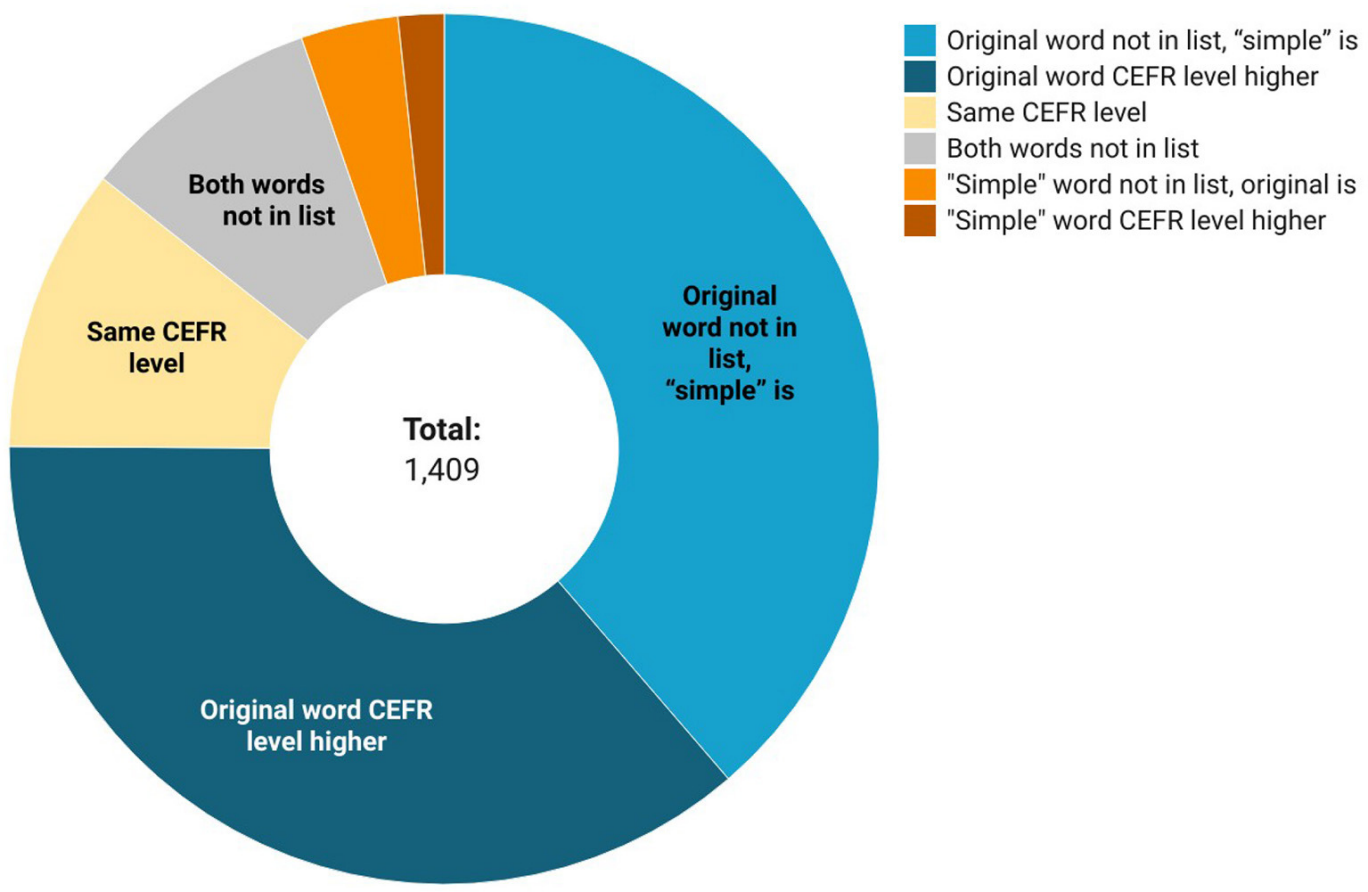
Word grade levels statistics.

There were 686 pairs where both words could be found in the CEFR vocabulary lists. Of them, in 513 cases the source word CEFR level was higher. In 545 cases the source word is not presented in the CEFR-graded lists while the target word is (see Figure 2). This means that in 75% of the cases the proposed simplified word is considered simpler by foreign language acquisition specialists, which shows that in most cases, the source word is indeed more complicated and less often used than the target word. Of particular interest is word pairs where the source and target words have the same CEFR level tags. Most of these cases can be explained as the choice of a word whose derivative appeared on the lists earlier, so the reader is more likely to guess its meaning (e.g., сердиться [to be grumpy] is replaced by злиться [to be angry]; both verbs are B2 level, but the cognate adjective злой [angry ADJ] appears at the earlier A2 level). In isolated cases where the target word has a higher CEFR level than the source word, the word choice might have been prompted by the desire to use and international synonym (e.g., расстояние [spacing, distance] → дистанция [distance]), as well as illustrating imperfections in possible vocabulary lists or human errors during text adaptation.

As word frequency is commonly used for identifying word complexity status in lexical simplification studies (Al-thunayyan and Azmi, 2021), we compared the IPM values (instances per million words) of word pairs, positing that the IPM of the source word in a pair should be lower than the IPM of the target word. We used a frequency dictionary of modern Russian language for this purpose (Lyashevskaya and Sharov, 2009).

**Figure 3:**
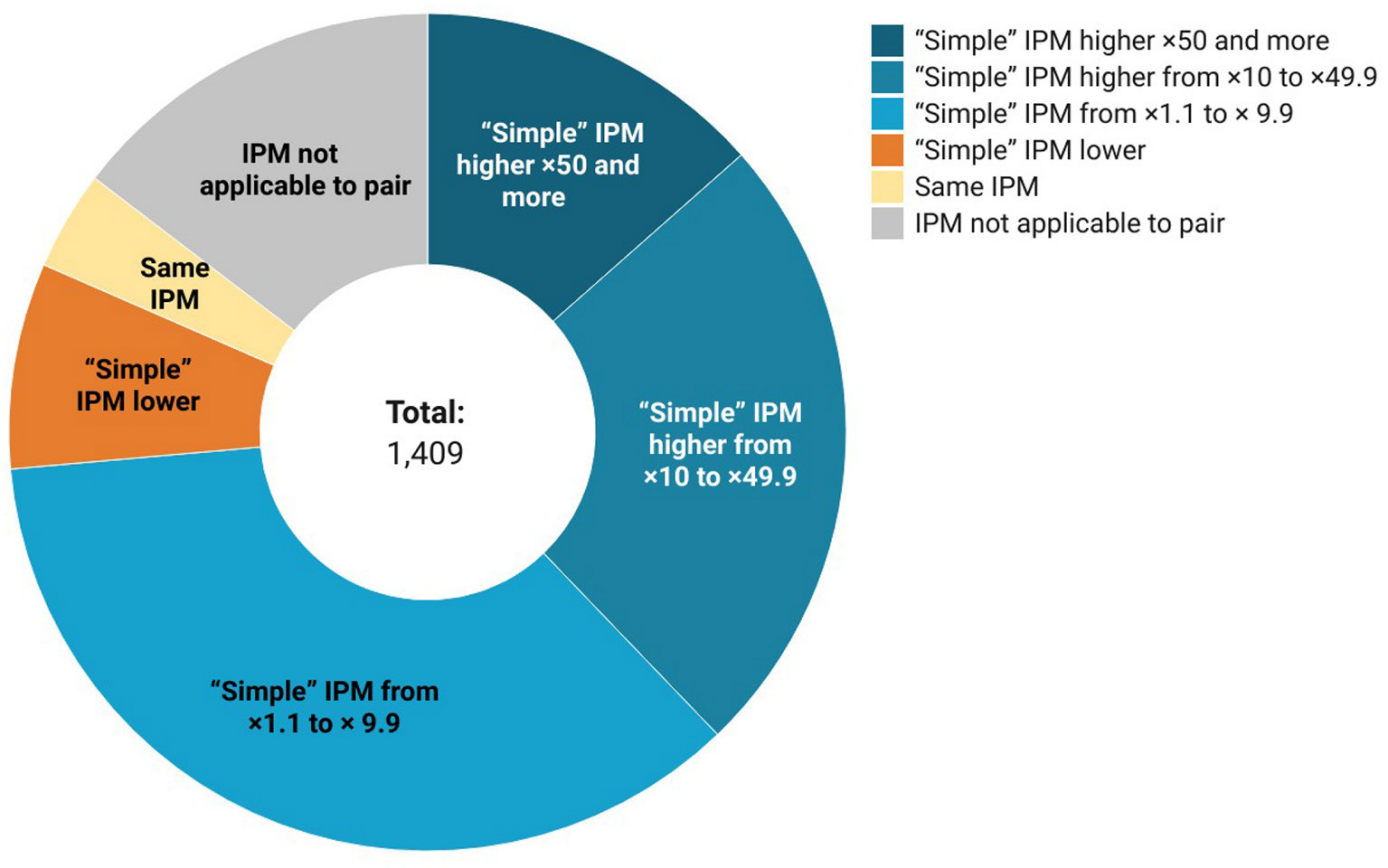
Frequency statistics.

In terms of frequency, we found out that of the 1,203 pairs where both source and target words were present in the chosen frequency dictionary, in 1,037 pairs the target IPM was higher than the source, in 112 pairs the source IPM was higher, and in 54 cases the IPMs were equal (see Figure 3). IPM is equal mostly in cases where the source word has a non-modern spelling (несчастие/несчастье), because in such cases source and target are lemmatized the same way.

The evaluations show that in most cases the “simpler” candidate from the list indeed appears earlier in a graded vocabulary list for language learners and/or is more frequent in Russian language than the original, complex word. Contrariwise, cases were found where the word chosen by the authors for adaptation turned out to be less frequent (8.1% of words) or related to a higher level of CEFR (5%). These cases seem to demonstrate word selection criteria that are relevant to the foreign language learners (for example, the internationality of a word, the presence of a frequently used derivative) and are case for which it is potentially difficult to automate lexical substitution.

## Discussion

In this paper, we described the creation of a list of word alignments from parallel Russian simplification data. We used two automatic aligners and human post-editing in order to choose word pairs where the source and target words can be considered synonyms in most contexts or where the target word is the modern spelling version of the source word.

During our research, we found out that there do not seem to be many cases of actual single-word lexical simplification (i.e., synonym replacement) in adapted readers for Russian L2 learners. Despite using different aligners, in both cases <10% of all single-word alignments of different words ended up with a score of 2 and were included in a final list. We can hypothesize that in adapted literature, there is more lexical simplification at the phrase level than the word level, or that perhaps such phenomena cannot be fully captured without special word aligners for parallel simplification data.

The resulting list allows us, first, to explore lexical adaptation strategies that are relevant for L2 learners. Only 75% of the word pairs fit the classical criteria of word complexity, such as word frequency or CEFR level. In other cases, the choice of lexical substitution was explained as a choice in favor of international words or derivatives from simple frequent words. This indicates the need to take these features into account at future stages of automating the process of lexical simplification.

Another application of the resulting list is to improve the quality of the next iterations of the aligning process, since now we can use these word pairs as points where we expect lexical substitution.

In the future, we want to expand the scope of this research to phrase-level simplification and to use other datasets. We hope to gather enough data to create reliable lexical-simplification systems and tools for computer-assisted text adaptation. We also hope that in the future less human editing will be needed during the creation of other word lists, because it will be possible to use our word list to train models for automatic evaluation of word pairs.

## Data availability statement

The datasets presented in this study can be found at: https://github.com/Digital-Pushkin-Lab/RuAdapt_Word_Lists.

## Author contributions

AD: conception and design of the study, formulation of research goals and aims, data collection, literature review, aligners building and evaluation, and writing the article. AL: literature review, data analysis, interpretation of results, and writing the article. ML: conception and design of the study, coordination of the expert annotation process, interpretation of results, and writing the article. All authors contributed to the article and approved the submitted version.

## Funding

The article was prepared in full within the state assignment of Ministry of Education and Science of the Russian Federation for 2020–2024 (No. FZNM-2020-0005).

## Conflict of interest

The authors declare that the research was conducted in the absence of any commercial or financial relationships that could be construed as a potential conflict of interest.

## Publisher's note

All claims expressed in this article are solely those of the authors and do not necessarily represent those of their affiliated organizations, or those of the publisher, the editors and the reviewers. Any product that may be evaluated in this article, or claim that may be made by its manufacturer, is not guaranteed or endorsed by the publisher.

## Footnotes

^1^ https://github.com/Digital-Pushkin-Lab/RuAdapt

^2^ https://github.com/robertostling/eflomal

^3^ https://www.nltk.org/_modules/nltk/translate/phrase_based.html

^4^ https://github.com/AlexeySorokin/NeuralMorphemeSegmentation

^5^Available at: https://github.com/Digital-Pushkin-Lab/RuAdapt_Word_Lists

^6^ https://stanfordnlp.github.io/stanza/
